# Recognition ability of untrained neural networks to symbolic numbers

**DOI:** 10.3389/fninf.2022.973010

**Published:** 2022-09-21

**Authors:** Yiwei Zhou, Huanwen Chen, Yijun Wang

**Affiliations:** The School of Automation, Central South University, Changsha, China

**Keywords:** symbolic number, number sense, spiking neural network, lateral inhibition, visual recognition

## Abstract

Although animals can learn to use abstract numbers to represent the number of items, whether untrained animals could distinguish between different abstract numbers is not clear. A two-layer spiking neural network with lateral inhibition was built from the perspective of biological interpretability. The network connection weight was set randomly without adjustment. On the basis of this model, experiments were carried out on the symbolic number dataset MNIST and non-symbolic numerosity dataset. Results showed that the model has abilities to distinguish symbolic numbers. However, compared with number sense, tuning curves of symbolic numbers could not reproduce size and distance effects. The preference distribution also could not show high distribution characteristics at both ends and low distribution characteristics in the middle. More than half of the network units prefer the symbolic numbers 0 and 5. The average goodness-of-fit of the Gaussian fitting of tuning curves increases with the increase in abscissa non-linearity. These results revealed that the concept of human symbolic number is trained on the basis of number sense.

## Introduction

Neurons in the inferotemporal cortex prefer basic shapes. They could selectively respond to certain objects and maintain this preference when the size and position of objects change (Tanaka, 2003; Cao et al., 2020). Some of the basic shapes are very similar to symbolic numbers, such as star, a figure-of-eight, and T junctions. Their combination could distinguish most shapes, such as Arabic numerals, letters, and words (Tsunoda et al., 2001; Tanaka, 2003). This ability is selected in the process of biological evolution and reflects the evolutionary history of the visual system (Mccandliss et al., 2003). Therefore, the brain may have the ability to distinguish symbolic numbers and non-symbolic numerosities without training.

Humans are not born to understand the meaning of symbolic numbers (Sella and Lucangeli, 2020). From a cognitive neuroscience perspective, the brain’s response to symbolic numbers should be expected to change over the course of learning and development as children go from initially perceiving symbolic numbers as meaningless shapes or sounds to having a rich representation of their meanings. This phenomenon is often referred to as “cultural brain plasticity” (Rathé et al., 2019, 2020). However, before the brain could understand the meaning of symbolic numbers, symbolic numbers must first be perceived and distinguished (Ansari, 2016). Studies on preschool children pointed out that children around the age of 2 years have not yet fully understood the meaning of symbolic numbers but only regard these numbers as a group of unexplained symbols (Suggate et al., 2018; Verschaffel et al., 2020). After 1–2 years of learning, children can deal with the meaning of symbolic numbers (Nisan and Kiziltepe, 2019; Bugden et al., 2021; Vogel and Smedt, 2021). At present, extensive studies have been conducted on how the trained brain represents symbolic numbers (Ferres-Forga and Halberda, 2020; Sokolowski et al., 2021a,b). However, how it distinguishes between different symbolic numbers before training remains to be studied.

Many models are dedicated to explaining the brain’s recognition of symbolic numbers (Shamim et al., 2018; Ali et al., 2019; Ahlawat et al., 2020; Chychkarov et al., 2021; Khanday and Dadvandipour, 2021; Mitani et al., 2021). Verguts and Fias (2004) believe that symbolic numbers and non-symbolic numerosities were converted into internal location codes through different paths, thus explaining the cause of the distance effect when comparing different symbolic numbers. The triple-coding model proposed by Dehaene and Cohen (1995) assumed that quantity processing may adopt three different representation systems and predicted a brain region specialized in processing symbolic numbers in the ventral visual stream. These two models explain the discriminant process of symbolic numbers from a biological point of view. However, researchers did not consider whether the model could have the asemantic processing abilities of symbolic numbers without training. In addition, many models were built by traditional artificial neural networks, such as convolutional neural networks, support vector machines, and k-nearest neighbor models (Grover and Toghi, 2018; Shamim et al., 2018; Hossain and Ali, 2019; Tahir and Pervaiz, 2020; Chychkarov et al., 2021; Khanday and Dadvandipour, 2021; Mitani et al., 2021). The experimental results of these models are difficult to explain from the perspective of biophysics. The structure of some models is complex, including multiple convolution layers and pooling layers; thus, explaining the symbolic number recognition process is difficult (Hossain and Ali, 2019; Ahlawat et al., 2020; Mitani et al., 2021). Therefore, using the spiking neural network model with biophysical significance is necessary to investigate whether and how the untrained model distinguishes symbolic numbers.

A spiking neural network based on biological interpretability was constructed in this work to explore whether untrained animals could distinguish different symbolic numbers and compare the difference between non-symbolic numerosity recognition and symbolic number recognition. This model and the previously constructed number sense model (Zhou et al., 2022) consist of a two-layer neural network and have lateral inhibition. The difference is that the model proposed in the present work belongs to the spiking neural network. The LIF neuron based on current was used as the neural network unit. In addition, lateral inhibition was achieved by negative connection weights. Therefore, the information processing process of the model has a strong biological basis. Under the condition of randomly setting the network connection weight without adjustment, the symbolic number dataset was inputted into the model to investigate the recognition abilities of the untrained model.

## Materials and methods

### Stimulus datasets

A non-symbolic numerosity dataset mimicking the dataset of Nasr et al. (2019) was constructed to test whether the model could reproduce the non-symbolic numerosity recognition abilities that animals had prior to training. The dataset consisted of 150 images. The number of items in the image was between 1 and 5. Therefore, each non-symbolic numerosity was represented by 30 different images. Each image in the stimulus set contained 28×28 pixels, and the stimulus intensity of each pixel ranged from 0 to 1. Each item was a circle with an area of 25 pixels.

**FIGURE 1:**
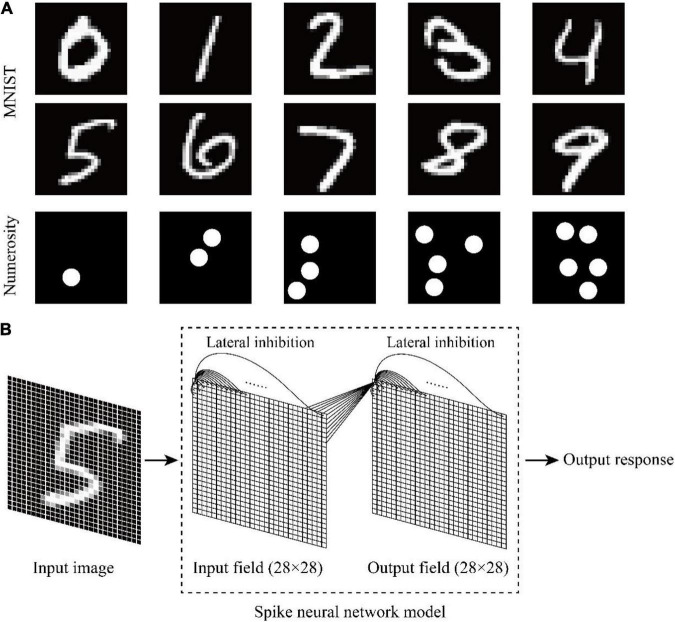
Schematic of the dataset and neural network structure. **(A)** Symbolic number dataset MNIST and non-symbolic numerosity dataset. **(B)** Two-layer spike neural network model with lateral inhibition.

The symbolic number dataset MNIST was used to investigate whether and how untrained animals recognize symbolic numbers. Symbolic numbers 0–9 were inputted into the model, and the output response of the model was observed. MNIST and the non-symbolic numerosity dataset are shown in Figure 1A.

### Spike neural network model

The programming language Python was used on the open-source machine learning platform Bindsnet to build a two-layer spike neural network model with lateral inhibition (Figure 1B). The input layer represented the visual pathway from the retina to the occipital lobe, and the output layer represented the visual pathway from the occipital lobe to the temporal lobe. The network size of the input layer and the output layer was 28×28. Each unit consisted of a current-based LIF neuron. The default parameters of current-based LIF neuron are shown in Table 1. Each neuron of the input layer corresponded to one pixel of the input image. The units located in different layers were fully connected, and initial weights followed the Gaussian distribution of μ=0.5 and σ^2^=0.1. Each time a new image was inputted, the connection weights between different layers were randomly generated without adjustment. Lateral inhibition was added to the model because it presented in primary visual cortex (Lu and Zuo, 2017) and neocortex (Zhou and Yu, 2018) associated with visual processing. The neurons in the same layer are connected with each other, and the weight is negative. The weight is also related to the distance between neurons. When the Euclidean distance between neurons increases, the weight decreases, and the mutual inhibition ability between neurons decreases.

**TABLE 1:** Default parameters of current-based LIF neuron.

Node parameters	Default	Description
traces	False	Whether to record spike traces
traces_additive	False	Whether to record spike traces additively
tc_trace	20.0	Time constant of spike trace decay
trace_scale	1.0	Scaling factor for spike trace
thresh	−52.0	Spike threshold voltage
rest	−65.0	Resting membrane voltage
reset	−65.0	Post-spike reset voltage
refrac	5	Refractory (non-firing) period of the neuron
tc_decay	100.0	Time constant of neuron voltage decay
tc_i_decay	2.0	Time constant of synaptic input current decay
lbound	None	Lower bound of the voltage

The weight between neurons in the same layer is expressed as follows:


(1) $$w_{x\,y}=-e^{\frac{-R_{x\,y}}{2\times \alpha^{2}}}$$

where *w*_*xy*_ is the weight between neuron *x* and neuron *y* in the same layer of neural network, *R*_*xy*_ is the Euclidean distance between two neurons, and α is the standard deviation of the Gaussian function. An increase in α indicates that the range of lateral inhibition of neurons increases. Although each layer of the model only performs lateral inhibition once, it reflects the result of multiple lateral inhibitions at different levels of the visual pathway. Comparison of the simulation results and experimental data (Kutter et al., 2018) revealed that the fitting degree between the simulation results and experimental data was high when the standard deviation of the input layer was α_*input*_ = 10 and that of the output layer was α_*output*_ = 20.

The stimulation duration of each image in the dataset was 2 s. Each pixel of image generates a certain frequency impulse sequence in accordance with pixel value to stimulate input layer neuron. The probability of impulse generation per millisecond follows the Bernoulli distribution.


(2) $$f\left(x|p\right)=\left\{ \begin{array}{c} p^{x}\,q^{1-x},x=0,1\\ 0,x\neq 0,1 \end{array},\right.$$

where the parameter *p* is 0.2 times the pixel value, and *x* = 1 indicates that pixel generates an impulse to stimulate the corresponding input layer neuron at the current time.

### Model response detection

Non-symbolic numerosity dataset was inputted into the model to test whether the model could reproduce the number sense that humans have without training. The average response frequency of each output layer neuron was recorded when the same non-symbolic numerosities were inputted, and the tuning curve of each neuron was obtained. If the tuning curve of a neuron contained the maximum response to a certain numerosity, then the neuron preferred the numerosity. Tuning curves with the same preference were averaged to obtain the average tuning curve of each non-symbolic numerosity. A brain study showed that the tuning curves of numerosity-selective neurons are more symmetrical on the logarithmic scale than on the linear scale (Kutter et al., 2018). Therefore, we drawn the average tuning curves of the model on the linear and logarithmic scales, and compared the symmetry of the curves to prove that the model can reproduce this characteristic. Moreover, the Gaussian function was fitted to the average tuning curves plotted on the linear scale and three non-linearly scales $\left(f\,\left(x\right)=x,f\,\left(x\right)=x^{\frac{1}{2}},f\,\left(x\right)=x^{\frac{1}{3}},f\,\left(x\right)={\text{log}}_{2}\,\left(x\right)\right)$ to quantitatively investigate the symmetry of the average tuning curves under non-linearly scales. A bar graph was used to plot the average goodness-of-fit (r-square) of the Gaussian function to the average tuning curves on different scales to verify whether the average goodness-of-fit increase with the increase in abscissa non-linearity. A scattergram was used to plot the bandwidth of the Gaussian function to the average tuning curves on different scales to verify whether the bandwidth remain unchanged in abscissa non-linearity. Biological experiment also showed that the frequency distribution of the preferred numerosities of numerosity-selective neurons conforms to the distribution characteristics of high at both ends and low in the middle (Kutter et al., 2018). A bar graph was used to plot the frequency distribution of the preferred numerosities of numerosity-selective neurons of the model to verify whether the frequency distribution of the model conforms to this characteristic.

The MNIST dataset was inputted into the model after confirming that the model could simply simulate the non-symbolic numerosity information processing process of the ventral visual stream. The purpose is to explore whether untrained animals could distinguish different symbolic numbers and compare the difference between non-symbolic numerosity recognition and symbolic number recognition. The responses of neurons with the same preference at the input of the MNIST dataset was averaged to obtain the average tuning curves. The characteristics of curves on linear and logarithmic scales were then compared. The frequency distribution of the preferred number of number-selective neurons was computed. The goodness-of-fit and standard deviation of the Gaussian function to the average tuning curves on four scales were also calculated.

## Results

### Non-symbolic numerosity recognition of model

**FIGURE 2:**
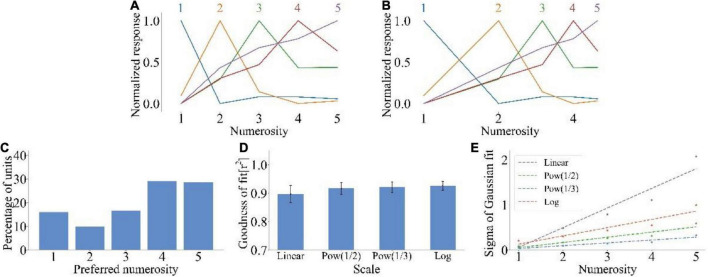
Output response of spike neural network model under non-symbolic numerosity dataset. **(A)** Average tuning curves for network units that prefer each non-symbolic numerosity plotted on a linear scale. The horizontal axis is the numerosity in the image, and the vertical axis is the average response after normalization. **(B)** Average tuning curves for network units that prefer each non-symbolic numerosity plotted on a logarithmic scale. The horizontal axis is the numerosity in the image and plotted on a logarithmic scale of *f*(*x*) = *log*_2_(*x*). **(C)** Distribution of preferred numerosities of numerosity-selective network units. The horizontal axis is the numerosity, and the vertical axis is the proportion of the number of units that prefer a specific numerosity in the total number of units. **(D)** Average goodness-of-fit measure for fitting Gaussian functions to tuning curves on different scales. The average response curves with the preferred numerosity ranging from 1 to 5 were combined via Gaussian fitting, and the goodness of fit was calculated using the four scales $\left(f\,\left(x\right)=x,f\,\left(x\right)=x^{\frac{1}{2}},f\,\left(x\right)=x^{\frac{1}{3}},f\,\left(x\right)=l\,o\,g_{2}\,\left(x\right)\right)$. **(E)** Standard deviation of the Gaussian function with an optimal fit for each tuning curve of numerosity-selective network units on different scales. The horizontal axis is the preferred numerosity, and the vertical axis is the standard deviation.

We inputted the non-symbolic numerosity dataset into the model to test whether the model could reproduce the number sense that humans have without training. Figure 2A shows the average tuning curve of neurons that preferred different non-symbolic numerosities. The neuron produced the maximum impulse response frequency when the input numerosity was equal to the preferred numerosity. A large distance between input and preferred numerosities indicated a small response of the neuron. The impulse response frequency of neurons decreased as the distance between the input numerosity and the preferred numerosity increased. The impulse response frequency of neurons with a larger preferred numerosity decreased more slowly when the number of items deviated from the preferred numerosity. Therefore, the average tuning curves of the model could reproduce size and distance effects observed in biological experiments related to number sense (Merten and Nieder, 2009; Kutter et al., 2018). The average tuning curves were more symmetrical on the logarithmic scale (Figure 2B) than on the linear scale (Figure 2A). Figure 2C shows that most neurons preferred the non-symbolic numerosities 1 and 5 (15.97 and 28.93%), indicating the distribution characteristics of high at both ends and low in the middle. Figure 2D shows that the average goodness-of-fit of the Gaussian fitting of the average tuning curves increased with the increase in abscissa non-linearity $\left(r_{\text{linear}}^{2}=89.72\%,r_{\text{log}}^{2}=92.65\%\right)$. Figure 2E illustrates that the standard deviation of Gaussian function increased with the increase in the preferred numerosity on the linear scale. On the contrary, the standard deviation of Gaussian function of other non-linear scales was nearly unchanged. The consistency between these results and the experimental data of human medial temporal lobe (Kutter et al., 2018) and monkey prefrontal cortex (Nieder and Merten, 2007) indicated that the model could simply simulate the non-symbolic numerosity information processing process of the ventral visual stream.

### Literature review

Animals can distinguish symbolic numbers. A female chimpanzee named “Ai” could associate symbolic numbers 1–6 with non-symbolic numerosities (Matsuzawa, 1985). Monkeys could associate 26 different symbolic numbers with the number of items from 0 to 25 (Livingstone et al., 2014) and reproduce size and distance effects when comparing symbolic numbers 1–4 (Diester and Nieder, 2010). In fact, birds could also produce abilities to distinguish symbolic numbers. A gray parrot named “Alex” could understand the Arabic numerals 0–8 (Pepperberg, 2013). Pigeons could respond selectively to symbolic numbers 1–6 (Xia et al., 2000). These experiments proved that the trained animals could distinguish symbolic numbers. Some researchers have also studied the brain’s response to symbolic numbers before training. Holloway et al. (2013) used an fMRI adaptation paradigm to examine the neural response to Hindu-Arabic numerals and Chinese numerical ideographs in a group of Chinese readers who could read both symbol types and a control group who could read only the numerals. They found that the control group who could read only the numerals also had a slight response to Chinese numerical ideographs (Holloway et al., 2013). Shum et al. (2013) also found that foreign symbolic numbers could activate the inferior temporal gyrus. These biological experiments showed that untrained animals could respond to symbolic numbers. In the present work, a model was constructed to explore whether untrained animals could distinguish different symbolic numbers, and the difference between non-symbolic numerosity recognition and symbolic number recognition was compared.

### Symbolic number recognition of model

**FIGURE 3:**
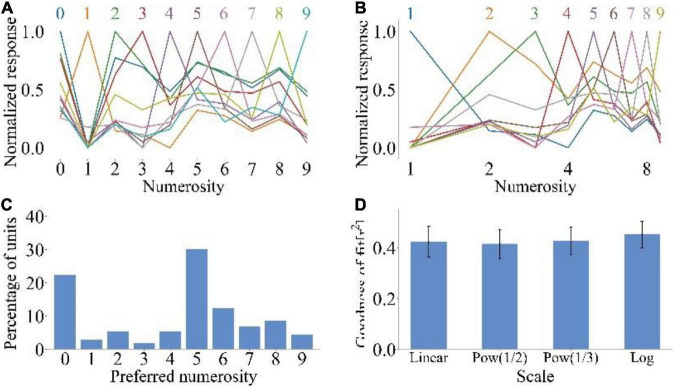
Output response of spike neural network model under symbolic number dataset MNIST. **(A)** Average tuning curves for network units that prefer each symbolic number plotted on a linear scale. **(B)** Average tuning curves for network units that prefer each symbolic number plotted on a logarithmic scale. **(C)** Distribution of preferred numbers of number-selective network units. **(D)** Average goodness-of-fit measure for fitting Gaussian functions to tuning curves on different scales.

We inputted the MNIST dataset into the model to explore whether untrained animals could distinguish different symbolic numbers. Figures 3A,B show the average tuning curves of neurons to symbolic numbers at linear and logarithmic scales, respectively. The untrained model could distinguish the symbolic numbers 0–9. Neurons that prefer specific symbolic numbers could also respond to other symbolic numbers, because in the symbolic number sequence, the symbolic numbers with close distance are more similar in form, and the physical similarity could affect the response of the model to the symbolic numbers (Cohen et al., 2013). However, compared with Figures 2A,B, the response of neuron did not show a downward trend when the input symbolic number was far away from the preferred symbolic number. Therefore, the average tuning curves of symbolic numbers could not reproduce size and distance effects. Figure 3C shows that the preferences of neurons covered all symbolic numbers contained in the MNIST dataset. The neurons preferring 0 and 5 were the most, accounting for 22.32 and 30.10%, respectively. When the range of symbolic numbers is 0–5, the preference distribution could show high distribution characteristics at both ends and low distribution characteristics in the middle. This is the same as the preference distribution of neurons when non-symbolic numerosity dataset is input. But when the range of symbolic numbers is 0–9, the symbolic number preference distribution of neurons (Figure 3C) differed from the non-symbolic numerosity preference distribution (Figure 2C). Because the feature extracted by lateral inhibition was different when the symbolic number and non-symbolic numerosity were inputted. The former extracted the quantitative features of items, while the latter extracted the edge features of symbolic numbers. The average goodness-of-fit of the Gaussian fitting of the average tuning curves increased with the increase in abscissa non-linearity $\left(r_{\text{linear}}^{2}=42.33\%,r_{\text{log}}^{2}=45.22\%\right)$. This is the same as the Gaussian fitting result of the average tuning curves when non-symbolic numerosity dataset is input (Figure 2D). All these results showed that untrained animals could distinguish symbolic numbers.

## Discussion

In the past few years, the investigation in the field of symbolic number recognition has mainly focused on deep learning technology. Convolutional neural network can automatically extract different features. Thus, it is often used to solve the problem of symbolic number recognition. The recognition accuracy of some CNN models is as high as 98 or 99% (Jarrett et al., 2009; Ciresan et al., 2011; Ciregan et al., 2012). Combining support vector machine with CNN model could achieve 99% recognition accuracy (Niu and Suen, 2012). However, the traditional symbolic number recognition system needs pre-training to achieve feature extraction and classification. Moreover, the focus of these studies is mainly on the improvement of the model parameters, super parameters, and SGD optimization algorithm to the recognition performance. No biophysical explanation of the brain’s information processing process of symbolic numbers is available. Verguts and Fias (2004) proposed a model to explain the brain’s recognition of symbolic numbers. In this model, symbolic numbers and non-symbolic numerosities were converted into internal location codes through different paths. Semantics of symbolic numbers are learned by presenting symbolic numbers and non-symbolic numerosities, thus explaining the cause of the distance effect when comparing different symbolic numbers. The triple-coding model proposed by Dehaene and Cohen (1995) is a very influential neuropsychological model. It assumed that quantity processing may adopt three different representation systems and predicted a brain region specialized in processing symbolic numbers in the ventral visual stream. These two models explain the discriminant process of symbolic numbers from a biological point of view. However, they did not consider the abilities to distinguish symbolic numbers before training.

Compared with other symbolic number recognition models, the model proposed in this manuscript has three main advantages. First, the model was constructed to simulate the non-symbolic numerosity information processing process of the ventral visual stream from the perspective of biological interpretability. The model used lateral inhibition to process visual information and considered LIF neurons as neural network units. Second, the number of network layers was simplified. The model used a two-layer neural network to analyze the process of symbolic number and non-symbolic numerosity processing. Third, many models are trained to recognize symbolic numbers (Jarrett et al., 2009; Ciresan et al., 2011; Ciregan et al., 2012), making it difficult to judge whether semantic and asemantic processing abilities of symbolic numbers is the result of training. In the present work, the asemantic processing ability of the model were investigated without training. The results showed that the untrained spike neural network model could perform asemantic recognition of symbolic numbers 0–9. Compared with non-symbolic numerosity recognition, the tuning curves of symbolic numbers could not reproduce size and distance effects. When the range of symbolic numbers is 0–5, the preference distribution of neurons could show high distribution characteristics at both ends and low distribution characteristics in the middle. This is the same as the preference distribution of neurons when non-symbolic numerosity dataset is input. But when the range of symbolic numbers is 0–9, the preference distribution of neurons (Figure 3C) could not show high distribution characteristics at both ends and low distribution characteristics in the middle. More than half of the network units preferred the symbolic numbers 0 and 5. The average goodness-of-fit of the Gaussian fitting of the tuning curves also increased with the increase in abscissa non-linearity (Figure 3D). Studies have shown that humans (Kutter et al., 2018), monkeys (Diester and Nieder, 2010) and pigeons (Xia et al., 2000) trained with symbolic numbers reproduce a distance effect in distinguishing symbolic numbers. The present work showed that the average tuning curves of the untrained model could not reproduce the distance effect, indicating that thus effect is not caused by the physical similarity of symbolic numbers (Cohen et al., 2013). This finding supported the hypothesis that human beings connect symbolic numbers with innate non-symbolic numerosity processing system through training to obtain the meaning of symbolic numbers. At present, some researchers are committed to studying the construction process of children’s symbolic number concept system (Suggate et al., 2018; Nisan and Kiziltepe, 2019; Verschaffel et al., 2020; Bugden et al., 2021; Vogel and Smedt, 2021). Their research results also support the idea that symbols acquire meaning by linking neural populations coding symbol shapes to those holding non-symbolic representations of quantities. Therefore, number sense may be the basis for the formation of human symbolic number concept and even arithmetic operation abilities.

Handwritten Arabic numeral dataset was used to investigate the symbolic number recognition abilities of untrained models. In fact, many kinds of symbolic number systems exist, such as Chinese numbers, Roman numbers, Sanskrit numbers, and Tamil numbers. Studying the similarity of the response of the model to different symbolic number systems is helpful to find out the reason why specific symbols are included in the symbolic number system. Studying the difference of the response of the model to different symbolic number systems is helpful to find out the reason why Arabic numerals are widely used. Therefore, investigating the recognition abilities of the model to other symbolic numbers is necessary. In addition, the effect of training on the abilities of animals to recognize symbolic numbers is not clear. STDP weight modification rule could be added in the future to spike the neural network model to compare the difference in the symbolic number processing abilities between the model before and after training.

## Data availability statement

The original contributions presented in this study are included in the article/supplementary material, further inquiries can be directed to the corresponding author.

## Author contributions

YZ contributed to the conceptualization, data curation, formal analysis, investigation, methodology, and writing of the original draft of the manuscript. HC contributed to the conceptualization and writing—review and editing of the manuscript. YW contributed to the writing—review and editing of the manuscript. All authors contributed to the article and approved the submitted version.
